# Protective Effects of Emodin on Lung Injuries in Rat Models of Liver Fibrosis

**DOI:** 10.1515/biol-2019-0069

**Published:** 2019-12-31

**Authors:** Lili Zhang, Huiying Zhang, Limin Wang, Yimin Fan, Cuiying Zhang, Xujiong Li, Dewu Han, Cheng Ji

**Affiliations:** 1 Department of Pathophysiology, Changzhi Medical College, No. 161, Jiefang East Street, Changzhi, Shanxi 046000, China; 2 Function Laboratory, Changzhi Medical College, Changzhi, Shanxi 046000, China; 3 Institute of Liver Diseases, Shanxi Medical University, Taiyuan, Shanxi 030001, China; 4 Research Center for Liver Diseases, Keck School of Medicine, University of Southern California, Loa Angeles, CA 90089, USA

**Keywords:** Emodin (EMD), Liver fibrosis, Hepatopulmonary syndrome (HPS), Lung injuries

## Abstract

**Objective:**

The aim of this study is to investigate the protective effects of emodin (EMD) on the lung injuries in the rat models of liver fibrosis.

**Methods:**

Liver fibrosis was established in rats and the effect of intervention using EMD treatment was determined. Liver and lung weight coefficients were measured and lung content of TNF-α (tumor necrosis factor α), MDA (malondialdehyde), NO (nitric oxide), and ONOO- (peroxynitrite) were determined. Finally, histopathological changes were evaluated.

**Results:**

Compared with the normal control group, the lung weight coefficient was significantly increased in the fibrosis model group. Moreover, pulmonary edema and inflammatory responses were observed. Levels of TNF-α, MDA, NO, and ONOO- in the lung homogenate were significantly increased in the fibrosis model group. After EMD treatment, the lung weight coefficients were significantly reduced. Moreover, pathological changes in the lung tissue were dramatically alleviated. Levels of TNF-α, MDA, NO, and ONOO- were significantly decreased.

**Conclusion:**

EMD exhibits protective effects against lung injuries in a rat model of liver fibrosis.

## 1 Introduction

Lungs play an important role in the maintenance of the body’s normal function. Under pathological conditions, the lungs become more vulnerable and susceptible to injuries. Hepatopulmonary syndrome (HPS), characterized by early onset and occult occurrence, is observed in early stages of acute or chronic liver diseases. The incidence of cirrhosis-induced HPS could be as high as 5%-32% [1]. Moreover, liver diseases are always accompanied by intestinal endotoxemia (IETM) [2]. Increased endotoxin activate the Kupffer cells to secret a large amount of pro-inflammatory cytokines, such as TNF-α (tumor necrosis factor α), which not only causes the degeneration and necrosis of hepatocytes, but also induces abnormal immune responses in the liver and promotes the fibrosis [3,4]. Lungs are the target organ for IETM, and the enhanced endotoxin stimulates the lung macrophages to produce and release large amounts of cytokines (mainly TNF-α), inducing the lung injuries and underlying the cirrhosis combined with HPS. Liver fibrosis is a common pathological process in various chronic liver diseases, and it may lead to development of cirrhosis. However, reports on lung injury and its severity in liver fibrosis patients are limited.

Emodin (EMD) is the main active monomer component in the traditional Chinese medicine of rhubarb, which has been shown to be able to inhibit hepatic inflammation and reduce liver injuries, exhibiting therapeutic effects on liver fibrosis. EMD is an effective inhibitor of the extracellular regulated protein kinases (ERK) and the P38 mitogen-activated protein kinases (P38MAPK) signaling pathways in the Kupffer cells, which could block the MAPK signaling pathway-mediated activation of liver Kupffer cells induced by LPS, and inhibit the endotoxin-stimulated TNF-α secretion [5]. Moreover, EMD could decrease intracellular Ca^2+^ concentration and down-regulate the activity of calmodulin kinase, thereby inhibiting the expression of cytokine-related genes, reducing the production of inflammatory mediators, and inhibiting the excessive inflammation [6]. Furthermore, EMD could reduce the permeability of the intestinal wall and capillaries, and significantly inhibit the proliferation of gut-associated bacteria, as well as the production and absorption of toxins in the intestines [7]. The anti-liver fibrosis effects of EMD and the related mechanisms have been well documented. However, the effects of EMD on the lung injuries in liver fibrosis in the clinic have not yet been fully investigated.

In this study, a rat model of liver fibrosis was established with compound pathogenic factors. The pathological changes in the lung tissue were observed. Moreover, the effects of EMD on the liver fibrosis-induced lung injuries and the possible mechanisms were investigated.

## 2 Materials and methods

### 2.1 Animals

Twenty adult male SD rats, weighing 200-220 g, were purchased from the Beijing Academy of Military Sciences, Beijing, China. They were kept in standard conditions with free access to food and water.

**Ethical approval**: The research related to animals use has been complied with all the relevant national regulations and institutional policies for the care and use of animals. The study protocol was approved by the animal experimental ethics committee of Changzhi Medical College. All procedures were in accordance with Ethical Issues in Animal Experimentation issued by Changzhi Medical College, the international guidelines and the 3R principles (refinement, reduction and replacement).

### 2.2 Animal grouping and model establishing

SD rats were randomly divided into the following four groups (n = 5): 1) the normal control group (serving as negative control); 2) the liver fibrosis model group (serving as positive control); 3) the low-dose EMD (20 mg/kg) treatment group; and 4) the high-dose EMD (40 mg/kg) treatment group. During model establishment, rats in the liver fibrosis model and EMD treatment (low- and high-dose) groups were fed with corn (80% corn meal, 20% lard, 0.5% cholesterol, for the first 2 w; corn mixed with 0.5% cholesterol, for the next 2 w), and subcutaneously injected with the 40% CCl_4_ (Tianjin Fuyu Fine Chemical Co., Ltd., Tianjin, China) at 0.6 ml/100 g body weight (for the first time), followed by injections at 0.3 ml/100 g body weight, once every 4 d, for a total of 28 d. In the EMD treatment groups, EMD suspension was administered daily by gavage (Chengdu ConBon Biotech Co., Ltd., Chengdu, Sichuan, China; the EMD purity was 99%, which was prepared into suspension at different concentrations with 0.5% sodium carboxymethyl cellulose before use). In the normal control group, the rats were fed with normal diet. In the normal control and model groups, the rats received saline by gavage [1]. After 4 w of model establishment, rats were anesthetized with 10% chloral hydrate (0.3ml/100g) through intraperitoneal injection. Then, under deep anesthesia, rats were subjected to blood sample collection from abdominal aorta until death. After that, the liver and lung tissues were collected for the following experiments.

### 2.3 Liver and lung weight coefficient calculation

The rat liver and lungs were extracted and the liver and lung weight coefficients were calculated according to the following formulations: Liver coefficient = liver weight (g) / body weight (g) × 100%; and Lung coefficient = lung wet weight (g) / body weight (g) × 100%.

### 2.4 Plasma biochemical tests

The plasma biochemical tests were performed by Zhengzhou Jinyu Inspection Center (Zhengzhou, Henan, China). The levels of endotoxin, homocysteine, alanine aminotransferase (ALT), aspartate aminotransferase (AST), total bilirubin (TB), total cholesterol (TC), triglyceride (TG), hyaluronic acid (HA), laminin (LN), IV collagen (IV-C), and III procollagen (PCIII) were tested. At least three independent measurements were performed for each index.

### 2.5 Determination of lung biochemical parameters

Lung homogenate (10%) was prepared with an ultrasonic cell grinder. MDA (malondialdehyde) and NO (nitric oxide) levels in the lung homogenate were detected with commercially available kits (Nanjing Jiancheng Bioengineering Institute, Nanjing, Jiangsu, China) according to the manufacturer’s instructions. Protein contents were determined with the Coomassie brilliant blue method (Nanjing Jiancheng Bioengineering Institute). The TNF-α level was assessed with radioimmunoassay (RIA) by the Beijing Yinghua Biotechnical Institute, Beijing, China. Peroxynitrite (ONOO^-^) content in the lung tissue was analyzed with ELISA by the Shanghai Yuanye Biotechnology Co., Ltd., Shanghai, China). At least three independent measurements were performed for each index.

### 2.6 Pathological examination

Lung and liver lobes were extracted and embedded with paraffin, and then cut into sections. The sections were subjected to the HE and Van Gieson (VG) staining, and the pathogenic changes in the lung and liver tissues, as well as the liver collagen fiber hyperplasia, were observed under light microscope. Images were analyzed with the BI-2000 image analysis system (Chengdu TME Technology Co, Ltd, Chengdu, China), and the liver fibrosis index was calculated as the collagen fiber area divided by the total imaging area. Ten fields were randomly selected from each section for the calculation.

### 2.7 Pulmonary macrophage counting

The lung tissue was subjected to the HE staining. Ten fields in each section were randomly selected for the assessment of liver fibrosis index, and the averaged pulmonary macrophages in the pulmonary alveolar and interval space were counted at 400× magnification.

### 2.8 Statistical analysis

Data were expressed as mean ± SD. Statistical analysis was performed using SPSS 10.0 software. One-way ANOVA was used for group comparison, with the LSD-t test as the post hoc test. Pearson correlation analysis was performed. *P* < 0.05 was considered as statistically significant.

## 3 Results

### 3.1 General observation

**Figure 1:**
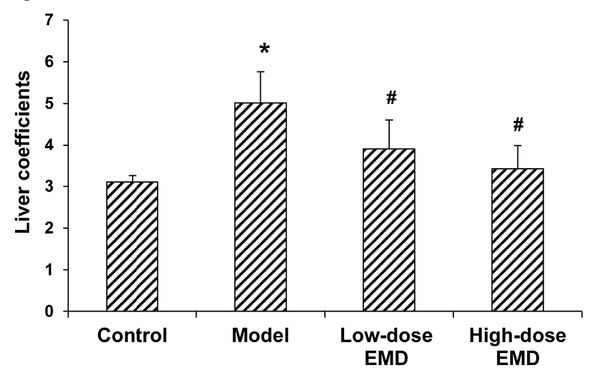
Comparison of rat liver weight coefficients in each group. Liver coefficients were calculated for the normal control, model, low-dose EMD (20 mg/kg), and high-dose EMD (40 mg/kg) groups. Compared with the normal control group, ^*^ *P* < 0.05; compared with the model group, ^#^ *P* < 0.05.

General observation showed that, in the normal control groups, the rats were lively and active, with supple and shiny fur and gradual body weight gain. Compared with the control rats, the rat models of liver fibrosis ate significantly less food, were lethargic, with arching spine, as well as ungroomed and dull fur, and body weight loss. However, in the EMD treatment groups, the behavioral and dietary activity were significantly improved, with yellow soft stool and yellow urine, and the body weights were slightly reduced compared with the model group. The liver weight coefficients for the groups were shown in Figure 1.

### 3.2 Plasma biochemical indexes

Plasma biochemical and liver fibrosis serological tests were then performed. The results showed that plasma levels of endotoxin, homocysteine, ALT, AST, TB, TC, and TG were significantly higher than the normal control group (*P* < 0.05), indicating that the compound pathogenic factors induced IETM in the rat models, with severely impaired liver cells, aggregation of liver lipids, metabolic disorders, and hyperhomocysteinaemia. Liver cell impairments suggested that the synthesis of albumin (ALB) was significantly impaired in the rat fibrosis models (*P* < 0.5). However, all biochemical markers for pathological change were significantly alleviated EMD-treated rats (*P* < 0.01). Moreover, plasma levels of ALT and AST in the high-dose EMD (40 mg/kg) group were significantly lower than those of the low-dose EMD (20 mg/kg) group (*P* < 0.05) (Table 1), indicating that the liver cell damage in the high-dose group was less severe than in the low-dose group.

**Table 1:** Changes in biochemical parameters in each group.

	Normal control group	Model group	Low-dose EMD (20mg/kg) group	High-dose EMD (40mg/kg) group
**Endotoxin (ng/mL)**	111.2±3.21	136.53±3.75^**^	115±4.17^##^	113.43±3.21^##^
**Homocysteine (nmol/mL)**	58.46±2.56	69.43±4.67^*^	62.21±0.72^#^	59.83±1.56^#^
**ALB (g/L)**	27.03±1.41	20.40±2.83^*^	26.73±0.40^#^	23.63±1.26^#^
**ALT (u/L)**	4.25±6.65	433.67±31.87^*^	158.75±17.81^#^	44.67±2.37^#Δ^
**AST (u/L)**	81.5±2.50	790.67±51.61^*^	351.0±22.31^#^	108.67±8.32^#Δ^
**TB (μmol/L)**	3.76±1.95	19.20±1.57^**^	6.73±1.58^##^	9.30±1.87^##^
**TC (mmol/L)**	1.51±1.72	2.12±0.18^*^	1.58±0.38^#^	1.74±0.17^#^
**TG (mmol/L)**	0.91±0.76	1.69±1.15^*^	1.21±0.17^#^	1.03±0.28^#^

Note: EMD, Emodin; ALT, alanine aminotransferase; AST, aspartate aminotransferase; TB, total bilirubin; TC, total cholesterol; TG, triglyceride. Compared with the normal control group, ^*^P< 0.01, ^**^P< 0.01; compared with the model group, ^#^*P*< 0.05, ^##^*P*< 0.01. Compared with EMD (emodin) 20 mg/kg group, ^Δ^P< 0.05.

The serological tests of the levels of HA, LN, IV-C, and PCIII reflect liver fibrosis. Our results showed that the levels of HA, LN, IV-C, and PCIII were significantly elevated in the fibrosis model groups than in the control group (*P* < 0.05), and the levels of these markers in the EMD treatment group were lower than the model group. Specifically, these four markers in the high-dose group were slightly lower than in the low-dose group (*P* > 0.05) (Table 2).

**Table 2:** Changes of HA, LN, IV-C, and PCIII contents in each group.

	Normal control group	Model group	Low-dose EMD (20 mg/kg) group	High-dose EMD (40 mg/kg) group
**HA (μg/L)**	96.37±9.80	168.08±3.46^*^	112.49±28.19^#^	98.46±5.94^#^
**LN (μg/L)**	29.3±1.36	47.2±7.08^*^	36.26±4.14^#^	32.61±1.58^#^
**IV-C (μg/L)**	2.42±0.53	3.31±0.41^*^	2.58±0.22^#^	2.54±0.17^#^
**PCIII (μg/L)**	1.56±0.50	6.91±1.86^*^	2.45±0.45^#^	1.68±0.60^#^

Note: HA, hyaluronic acid; LN, laminin; IV-C, IV collagen; PCIII, III procollagen. Compared with the normal control group, ^*^ *P* < 0.05; compared with the model group, ^#^ *P* < 0.05.

### 3.3 Liver pathology

**Figure 2:**
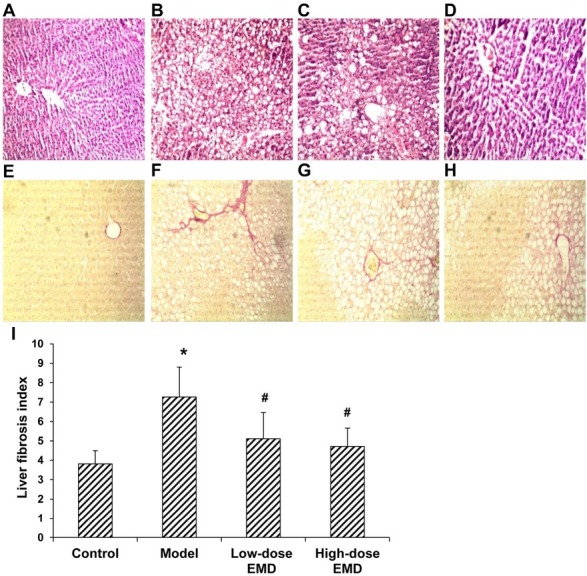
Detection of liver pathological changes and liver fibrosis in each group. (A-D) HE staining was performed to detect the pathological changes of the liver tissues from the normal control (A), model (B), low-dose EMD (20 mg/kg) (C), and high-dose EMD (40 mg/kg) (D) groups (100×). (E-H) VG staining was performed to detect the pathological changes of the liver tissues from the normal control (E), model (F), low-dose EMD (20 mg/kg) (G), and high-dose EMD (40 mg/kg) (H) groups (100×). (I) Comparison of liver fibrosis index in the rats of each group. Compared with the normal control group, ^*^ *P* < 0.05; compared with the model group, ^#^ *P* < 0.05.

The pathological changes in the rat livers were compared. The results showed normal lobular architecture in the normal control group, and the liver cells were radially arranged away from the central vein, with only a small amount of collagen fiber distributed in the portal area and around the central vein. In the model group, the hepatic cord was irregularly arranged, with evident hepatic steatosis and necrosis, and inflammatory cell infiltration. Moreover, Kupffer cell hyperplasia was observed, and the hepatic lobule was split by the collagen fibers. Deposition of a large amount of collagen fibers was noted in the portal area. In particular, in the low-dose EMD treatment group, the liver tissue structure was improved. There were mild necrosis in the lobular central area and mild inflammatory cell infiltration in the portal area, and the lobular tissue was split by the hyperplastic fibrous tissue. Moreover, in the high-dose EMD treatment group, integrate lobular architecture was noted, with significantly alleviated liver cell degeneration and necrosis, reduced fibrous connective tissue, and without obvious fiber interval (Figure 2).

### 3.4 Lung pathology

**Figure 3:**
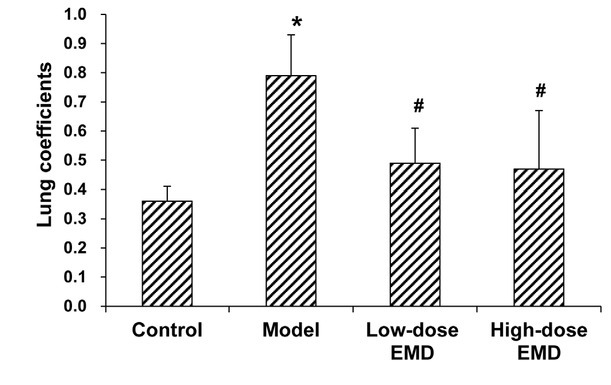
Comparison of rat lung weight coefficients in each group. Lung weight coefficients were calculated for the normal control, model, low-dose EMD (20 mg/kg), and high-dose EMD (40 mg/kg) groups. Compared with the normal control group, ^*^ *P* < 0.05; compared with the model group, ^#^ *P* < 0.05.

To investigate pathological changes in the lungs of rat models, the lung weight coefficients were calculated and the HE staining was performed. General observation showed that, in the normal control group, the lung tissue was pink, soft and shiny, with smooth surface. However, in the model group, both lung lobes were swollen, with significant congestion, and bleeding and necrotic lesions on the surface. On the other hand, in the EMD treatment groups, the observations were similar to the normal control group, except for relatively larger volumes. The lung weight coefficients could reflect edema in the lung tissue. Compared with the normal control group, the lung weight coefficients in the model group were significantly elevated (*P* < 0.05), suggesting significant lung edema in the rats with liver fibrosis. However, in both the EMD treatment groups, there was no obvious pulmonary edema, and no significant differences between the low-and high-dose groups (Figure 3).

**Figure 4:**
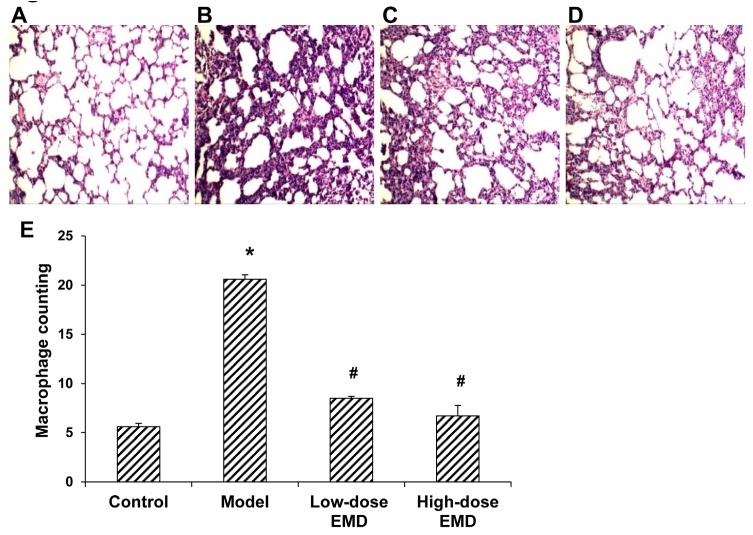
Pathological changes and macrophage counting in lung tissues in each group. (A-D) HE staining was performed to detect the pathological changes of the lung tissues (100×) from the normal control (A), model (B), low-dose EMD (20 mg/kg) (C), and high-dose EMD (40 mg/ kg) (D) groups. (E) Macrophage counting in lung tissues in each group. Compared with the normal control group, ^*^ *P* < 0.05; compared with the model group, ^#^ *P* < 0.05.

In addition, HE staining showed intact normal lung tissue in the normal control group, with even intervals, and no obvious inflammation or exudate in the pulmonary alveolar space. However, in the model group, narrowing alveolar lumen and thickening alveolar septa were observed, and there were plenty of macrophages and neutrophils accumulating in the pulmonary alveolar and the interval space. Low-dose EMD treatment significantly alleviated the inflammatory cells and exudate in the pulmonary alveolar space, and the pathological changes in the lung tissue. Moreover, high-dose EMD treated rats presented the slightest lung injuries with basically normal lung tissues and structures (Figure 4). The counting of pulmonary macrophages in the pulmonary alveolar and interval space showed that, compared with the normal control group, the pulmonary macrophages were significantly elevated in the model group (*P* < 0.05), indicating the obvious inflammatory responses in the lung tissue. However, compared with the fibrosis model group, the pulmonary macrophages were significantly reduced in the EMD treatment groups (*P* < 0.05). No obvious inflammatory responses were observed in the lung tissue, and no significant differences were observed between the low- and high-dose groups (Figure 4).

### 3.5 Lung homogenate detection and correlation analysis

Levels of TNF-α, MDA, NO and ONOO^-^ in the lung homogenate were significantly elevated in the model group compared to the normal control group (*P* < 0.05). Compared with model group, the levels of TNF-α, MDA, NO and ONOO^-^ in the lung homogenate were significantly reduced in the EMD treatment groups (*P* < 0.05), and no significant differences were noted between the low- and high-dose groups (*P* > 0.05) (Table 3).

**Table 3:** Contents of TNF-α, MDA, NO, and ONOO^-^ in each group.

	Normal control group	Model group	Low-dose EMD (20 mg/kg) group	High-dose EMD (40 mg/kg) group
**TNF-α (ng/mg)**	0.008±0.004	0.023±0.004^*^	0.012±0.007^#^	0.010±0.006^#^
**MDA (μmol/g)**	2.33±1.12	5.05±1.72^*^	2.63±1.46^#^	2.62±0.48^#^
**NO (μmol/g prot)**	0.13±0.03	0.74±0.28^*^	0.30±0.08^#^	0.38±0.19^#^
**ONOO- (μmol/g)**	0.70±0.20	1.42±0.65^*^	0.86±0.14^#^	0.89±0.09^#^

Note: EMD, Emodin; TNF-α, tumor necrosis factor α; MDA, malondialdehyde; NO, nitric oxide; ONOO^-^, peroxynitrite. Compared with the normal control group, ^*^ *P* < 0.05; compared with the model group, ^#^ *P* < 0.05.

In addition, correlation analysis showed that the plasma endotoxin level was positively related to the TNF-α level in the lung homogenate (*P* < 0.01). Moreover, the level of TNF-α in the lung homogenate was also positively correlated with the levels of MDA, NO, and ONOO^-^ (*P* < 0.05) (Table 4).

**Table 4:** Correlation analysis of the indexes in rat models of liver fibrosis.

Correlated indexes	r	P value
**Plasma level of endotoxin** vs **content of TNF-α in lung homogenate**	0.721	< 0.01
**Content of TNF-α** vs **content of MDA in lung homogenate**	0.573	< 0.05
**Content of TNF-α** vs **content of NO in lung homogenate**	0.623	< 0.05
**Content of TNF-α** vs **content of ONOO- in lung homogenate**	0.685	< 0.05

Note: EMD, Emodin; TNF-α, tumor necrosis factor α; MDA, malondialdehyde; NO, nitric oxide; ONOO^-^, peroxynitrite.

## 4 Discussion

In the present study, a rat model of liver fibrosis was successfully established with compound pathogenic factors. After 4 w, the rat serum and liver homogenate were subjected to various tests and pathological detections.

Histological detection showed that the lung septa were widened in the rat models with significantly increased alveolar exudate and macrophages in the pulmonary alveolar and interval space, and elevated lung coefficient. These results suggest the occurrence of pulmonary edema. Moreover, the levels of TNF-α, MDA, NO, and ONOO^-^ in the lung homogenate were significantly increased, suggesting that the liver fibrosis would cause the pathological changes in the lung tissue, mainly the inflammatory responses.

The plasma endotoxin levels were dramatically elevated in the fibrosis groups, suggesting the formation of IETM during liver fibrosis in these models. However, the treatments of EMD significantly reduced the plasma level of endotoxin in the rat models and alleviated IETM. The correlation analysis showed positive correlation between plasma endotoxin level and TNF-α level in the hepatic homogenate, and we suppose that IETM and TNF-α are two contributors for the lung pathological changes in rat models of liver fibrosis. Treatments of EMD dramatically alleviated the pathological changes in the lung tissue, indicating that the aggravated and mitigated hepatic pathology would directly affect the lung tissue. Therefore, the treatment of the pathological changes in the liver is essential for treating lung disorders. EMD could alleviate the IETM via various mechanisms, such as reduction of bacteria, endotoxin, and TNF-α in the circulation, indirectly protecting the lung tissue.

Under physiological conditions, there are only a relatively small amount of macrophages in the lung alveolar space. When there is inflammation or infection in the lung tissue, endotoxin attracts and activates macrophages to produce and release large amounts of inflammatory cytokines. The accumulation of a large number of macrophages in the lungs leads to excessive inflammation and thus aggravates the tissue damage [8,9]. Our results showed that the macrophages were significantly accumulated in the pulmonary alveolar and the interval space in the rat models of liver fibrosis, with elevated levels of TNF-α in the homogenate. TNF-α directly injures the lung tissue and promotes the aggregation and adhesion of neutrophils to endothelial cells, which then induces the pulmonary vascular endothelial cell injuries and increases the permeability of the capillary wall via releasing oxygen free radicals and lysosomal enzymes [10]. Moreover, TNF-α may further induce the accumulation and infiltration of pulmonary intravascular macrophages to increase the expression of the iNOS and eNOS and result in sustained release of NO [2]. High level of NO causes pulmonary vasodilation and lead to hypoxemia. NO is also involved in the rapid non-enzyme chemical reaction with the superoxide anion to produce peroxynitrite (ONOO^-^), which can stimulate the lipid peroxidation, leading to the cell membrane dysfunction, structural damage, and the oxidation of pulmonary surfactant-a stronger oxidant than NO and O_2_^-^ [2,11]. On the other hand, TNF-α can induce oxygen free radicals, which oxidizes the interstitial collagen-hyaluronic acid in the lungs, changes the interstitial stability, and aggravates the lung injuries [12].

Meanwhile, the level of lipid peroxidation product MDA in the lung homogenate elevates, which further amplifies the inflammatory responses, increases cellular damage and pulmonary edema. The correlation analysis in our study showed that the TNF-α level in the lung homogenate was positively correlated with the levels of MDA, NO, and ONOO^-^. Besides the IETM-induced lung injuries, the first pro-inflammatory cytokine TNF-α after LPS stimulation can further cause the release of various vasoactive substances and inflammatory mediators, enhancing the lung injuries. Our results showed that, compared with the model group, the lung weight coefficients in the EMD treatment groups were significantly lower, and the macrophages in the pulmonary alveolar and interval space under microscopy were obviously reduced. These results suggest that EMD can mitigate the pulmonary capillary injuries, reduce inflammatory exudate, and alleviate the pulmonary edema. Moreover, the treatments of EMD significantly decreased the levels of TNF-α, MDA, NO, and ONOO^-^ in the lung homogenate, and reduced the inflammatory responses in the lung tissue. We suggest possible mechanisms as: 1) EMD inhibits the release of TNF-α from pulmonary macrophages; 2) EMD strongly inhibits the activation of NF-κB, and attenuates the secondary cytokine cascade of the pro-inflammatory factors [5,13]; 3) EMD inhibits the activation of the iNOS gene and decreases the synthesis and release of NO in the inflammatory responses in the lung tissue[14]; and 4) EMD causes MDA level decline in the lung tissue, protects the activity of superoxide dismutase (SOD), and reduces the injuries of lung alveolar epithelial cell injuries induced by free radicals [1]. Therefore, we suppose that EMD might directly fight against inflammatory responses, scavenge oxygen free radicals, exhibit anti-oxidative effects in the lung tissue and alleviate the lung injuries induced by IETM in the case of liver fibrosis.

In the present study, the therapeutic effects of EMD at two doses on the lung injuries in rat models of liver fibrosis were investigated. Our results showed no significant differences on the effects of EMD on lung injuries between the two treatment doses, indicating that EMD may exert therapeutic effects over a wide treatment dose range. Further studies are warranted.

In conclusion, during the process of liver fibrosis, multiple mechanisms might be involved in the pathological changes in the lungs. EMD could not only indirectly reduce the liver fibrosis-induced lung injuries, but also directly mitigate the lung lesions. These findings might contribute to revealing the new mechanisms for HPS in clinic, and provide evidence for the development of effective non-surgical treatment for the disease.
